# A Comparison of Clinical and Epidemiological Characteristics of Fatal Human Infections with H5N1 and Human Influenza Viruses in Thailand, 2004–2006

**DOI:** 10.1371/journal.pone.0014809

**Published:** 2011-04-29

**Authors:** Vivek Shinde, Wanna Hanshaoworakul, James M. Simmerman, Ubolrat Narueponjirakul, Wiwan Sanasuttipun, Suchada Kaewchana, Darin Areechokechai, Kumnuan Ungchusak, Alicia M. Fry

**Affiliations:** 1 Influenza Division, Centers for Disease Control and Prevention, Atlanta, Georgia, United States of America; 2 Epidemic Intelligence Service, Centers for Disease Control and Prevention, Atlanta, Georgia, United States of America; 3 Bureau of Epidemiology, Thailand Ministry of Public Health, Nonthaburi, Thailand; 4 International Emerging Infections Program, Thailand Ministry of Public Health – United States Centers for Disease Control and Prevention Collaboration, Ministry of Public Health, Nonthaburi, Thailand; University of Texas Medical Branch, United States of America

## Abstract

**Background:**

The National Avian Influenza Surveillance (NAIS) system detected human H5N1 cases in Thailand from 2004–2006. Using NAIS data, we identified risk factors for death among H5N1 cases and described differences between H5N1 and human (seasonal) influenza cases.

**Methods and Findings:**

NAIS identified 11,641 suspect H5N1 cases (e.g. persons with fever and respiratory symptoms or pneumonia, and exposure to sick or dead poultry). All suspect H5N1 cases were tested with polymerase chain reaction (PCR) assays for influenza A(H5N1) and human influenza viruses. NAIS detected 25 H5N1 and 2074 human influenza cases; 17 (68%) and 22 (1%) were fatal, respectively. We collected detailed information from medical records on all H5N1 cases, all fatal human influenza cases, and a sampled subset of 230 hospitalized non-fatal human influenza cases drawn from provinces with ≥1 H5N1 case or human influenza fatality.

Fatal versus non-fatal H5N1 cases were more likely to present with low white blood cell (p = 0.05), lymphocyte (p<0.02), and platelet counts (p<0.01); have elevated liver enzymes (p = 0.05); and progress to circulatory (p<0.001) and respiratory failure (p<0.001). There were no differences in age, medical conditions, or antiviral treatment between fatal and non-fatal H5N1 cases. Compared to a sample of human influenza cases, all H5N1 cases had direct exposure to sick or dead birds (60% vs. 100%, p<0.05). Fatal H5N1 and fatal human influenza cases were similar clinically except that fatal H5N1 cases more commonly: had fever (p<0.001), vomiting (p<0.01), low white blood cell counts (p<0.01), received oseltamivir (71% vs. 23%, p<.001), but less often had ≥1 chronic medical conditions (p<0.001).

**Conclusions:**

In the absence of diagnostic testing during an influenza A(H5N1) epizootic, a few epidemiologic, clinical, and laboratory findings might provide clues to help target H5N1 control efforts. Severe human influenza and H5N1 cases were clinically similar, and both would benefit from early antiviral treatment.

## Introduction

With the emergence of an epizootic of highly pathogenic avian influenza A(H5N1) virus among poultry in Southeast Asia in December 2003, Thailand's first patients with influenza A(H5N1) virus infection (hereafter referred to as H5N1 cases) were identified in January 2004 [1]. As part of Thailand's public health response, the National Avian Influenza Surveillance (NAIS) system was established to detect, investigate, and control H5N1 cases [1], [2]. In the setting of heightened concern over the potential emergence of a widespread influenza A(H5N1) epidemic among humans, many thousands of suspect H5N1 cases were investigated and 25 H5N1 cases were eventually confirmed; in addition, 18% of suspect H5N1 cases tested positive for a human (seasonal) influenza virus infection (hereafter referred to as human influenza cases) [3]. To identify possible risk factors or predictors of death due to influenza A(H5N1) infection and to describe clinical differences between H5N1 and human influenza cases during the Thailand H5N1 epizootic, we compared clinical characteristics of fatal H5N1 cases to non-fatal H5N1 cases, and characteristics of fatal H5N1 cases to fatal human influenza cases detected in the NAIS system.

## Methods

Thailand's National Avian Influenza Surveillance System (NAIS) was a laboratory-based surveillance system to detect (human) H5N1 cases. NAIS was established by Thailand's Ministry of Public Health (MOPH) in December 2003 following Thailand's first outbreaks of influenza A(H5N1) among poultry. Laboratory and epidemiological response components of the surveillance system have been previously described [1], [2], [3]. Surveillance for H5N1 cases was healthcare facility-based, involving all public and private hospitals, outpatient departments, and freestanding clinics in all provinces of the country. Surveillance case definitions and case management guidelines from the MOPH were disseminated to provincial and local public health offices and to health care facilities. Case definitions were adapted from official WHO guidelines [4]. A person presenting to medical care was considered to be a suspect H5N1 case if the person had a clinical presentation of fever and respiratory symptoms or evidence of pneumonia, and had at least one of the following high risk H5N1 virus exposures: 1) contact with sick or dead birds within 7 days prior to symptom onset, 2) residence in an area experiencing bird die-offs in the 14 days prior to symptom onset, or 3) contact with another suspect H5N1 case or person with pneumonia in the 10 days prior to symptom onset. After definitive virologic testing (described below), suspect H5N1 cases were determined to be: 1) a laboratory confirmed H5N1 case, or 2) a laboratory confirmed human influenza case (A/H3N2, A/H1N1, or type B), or 3) excluded for influenza infection.

Suspect H5N1 case detection and management occurred in the following way: once a suspect H5N1 case was identified by a clinician, the patient was isolated, diagnostic respiratory specimens were obtained, antiviral therapy with oseltamivir was initiated, local and provincial public health authorities were notified, and the patient was referred to a provincial or regional hospital for advanced medical care if clinically warranted [1]. Report of a suspect H5N1 case to public health authorities triggered an epidemiological investigation and a public health response by district, provincial, and or regional Surveillance and Rapid Response Teams (SRRTs) [1]. Respiratory specimens collected from suspect H5N1 cases—consisting of nasopharyngeal swabs, throats swabs, or sputum from endotracheal suction—were tested by a network of 13 regional laboratories and one central laboratory overseen by the Thailand National Institute of Health (Thailand NIH); diagnostic virologic testing consisted of conventional or real-time reverse-transcriptase polymerase chain reaction (RT-PCR) for highly pathogenic avian influenza A(H5N1) and human influenza viruses (A(H1N1), A(H3N2), and type B) [2]. Results of virologic testing were relayed back to the treating clinicians and public health authorities via direct notification and through an online web-based reporting system.

### Retrospective Cohort Study

**Figure 1:**
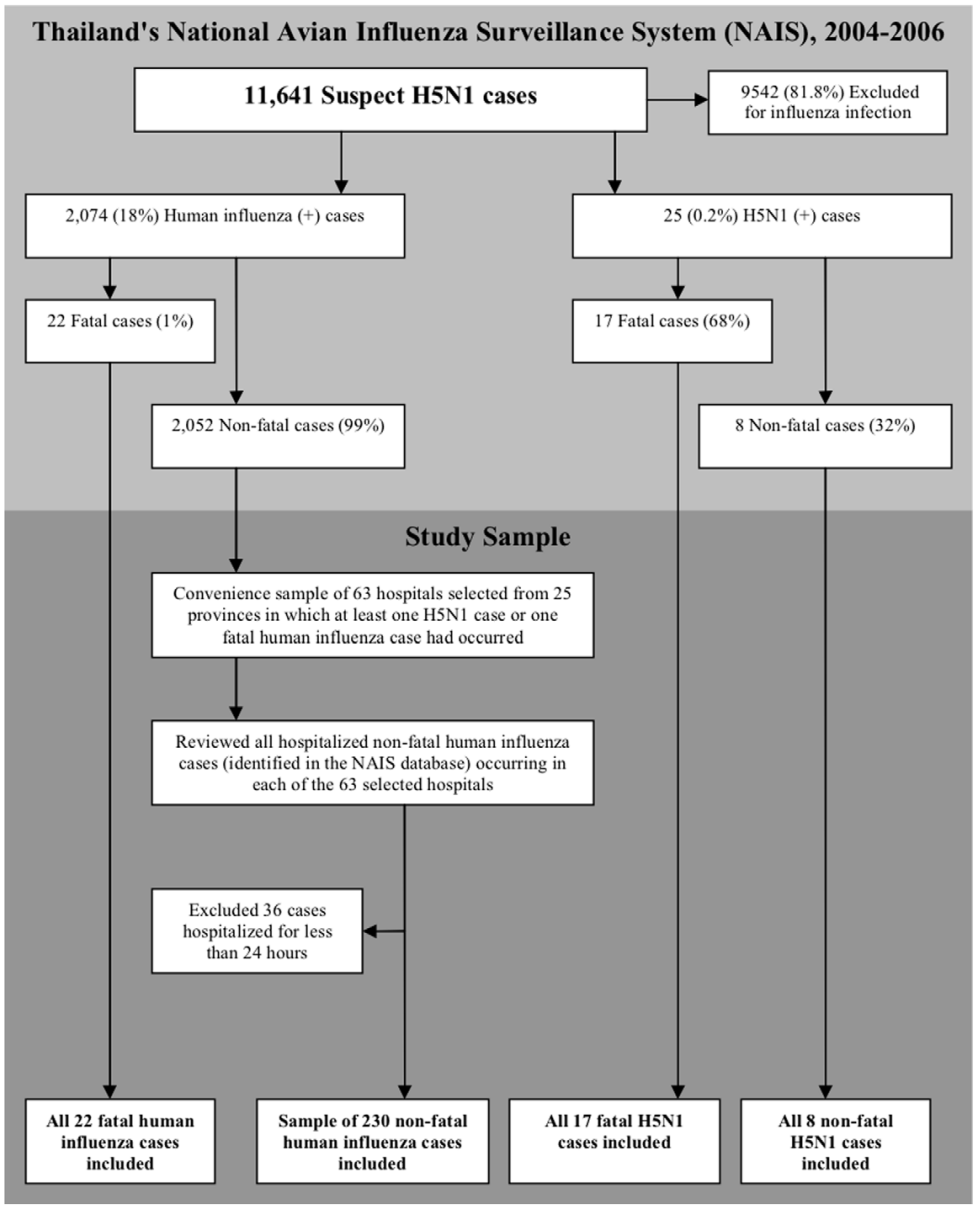
Algorithm describing the selection of the study sample from Thailand's National Avian Influenza Surveillance (NAIS) system.

We conducted a retrospective cohort study of H5N1 and human influenza cases captured in Thailand's NAIS system between January 2004 and December 2006. A description of the study design has been reported previously in a companion study [3]. Only patients with laboratory confirmed H5N1 or human influenza who were hospitalized for ≥1 day were eligible for inclusion in our study. The final study sample (Figure 1) consisted of the following three groups of patients: 1) all patients with fatal and non-fatal laboratory confirmed H5N1 infection; 2) all patients hospitalized with fatal laboratory confirmed human influenza infection; and 3) a sampled subset of patients hospitalized with non-fatal laboratory confirmed human influenza infection. Due to logistical and resource considerations, the latter sampled subset of hospitalized non-fatal human influenza cases was selected in a two-step process by: a) first selecting a convenience sample of 63 hospitals drawn from 25 of 76 Thai provinces in which at least one H5N1 case or human influenza case death had been reported); b) then we reviewed all hospitalized human influenza cases notified in the NAIS system that presented to each of the 63 selected hospitals. The sampling approach was designed to ensure that non-fatal human influenza cases were drawn from an epidemiologically similar population from which H5N1 and fatal human influenza cases arose.

Medical charts of sampled patients were reviewed by supervised teams of trained medical and public health professionals from the MOPH and the Thailand MOPH-U.S.CDC Collaboration (TUC) and a form was used to abstract detailed demographic, epidemiological, clinical, treatment, and outcome data from medical charts. Data were analyzed using STATA (Version 8, College Station, TX) statistical software. Disaggregated clinical and epidemiological data for 17 of 25 H5N1 cases have been previously reported, however, the data presented here represent a new abstraction and a complete analysis of all 25 H5N1 cases detected in Thailand during the study period [2], [5], [6]. The statistical analysis consisted of the following three parts: 1) a comparison of demographic characteristics and high-risk exposures between cases testing positive for H5N1 versus a sample of all human influenza cases; 2) a comparison of clinical characteristics associated with fatal and non-fatal outcome among H5N1 cases; and 3) a comparison of clinical characteristics of fatal H5N1 versus fatal human influenza cases. A previously published study compares patients with fatal and non-fatal human influenza from the NAIS system [3]. The denominators used to calculate proportions of patients with a given characteristic varied by the completeness of the available medical chart. Statistical comparisons of categorical variables were performed using Pearson's chi-square test or Fisher's exact test, whereas continuous variable were compared using the Kruskal-Wallis test. All *p*-values were two-sided, and considered statistically significant at a *p*- value <0.05. We used univariate logistic regression to estimate unadjusted odd ratios and 95% confidence intervals (CI) of a fatal outcome among H5N1 cases given a particular covariate, and also to determine the unadjusted odds ratios and 95% confidence intervals of a fatal H5N1 outcome versus a fatal human influenza outcome given a particular covariate.

This research was carried out with approval from and in compliance with the standards of the ethical review committees of the MOPH and the United States Centers for Disease Control and Prevention (US CDC). Informed consent was not required as data were analyzed anonymously.

## Results

### The National Avian Influenza Surveillance (NAIS) system and the study population

During January 2004 through December 2006, 11,641persons across Thailand were identified as suspected H5N1 cases in the NAIS system; among these, 2,074 (18%) persons in 73 of 76 Thai provinces tested positive for human influenza (A/H1N1, A/H3N2, or type B) and 25 persons in 19 of 76 provinces tested positive for H5N1 virus infection [3], [7]. Of the 2,074 human influenza cases, 22 (1%) were fatal and, of the 25 H5N1 cases, 17 (68%) were fatal (Figure 1) [3].

Our study included the following: all 25 H5N1 cases, all 22 fatal human influenza cases (arising from 15 of 76 provinces), and a sampled subset of 230 (of 2074 total) hospitalized non-fatal human influenza cases drawn from 25 of 76 Thai provinces in which at least one fatal H5N1 or human influenza case had occurred (Figure 1).

### Geography, seasonality, epidemiological exposures, and time interval to presentation of H5N1 and human influenza cases

**Figure 2:**
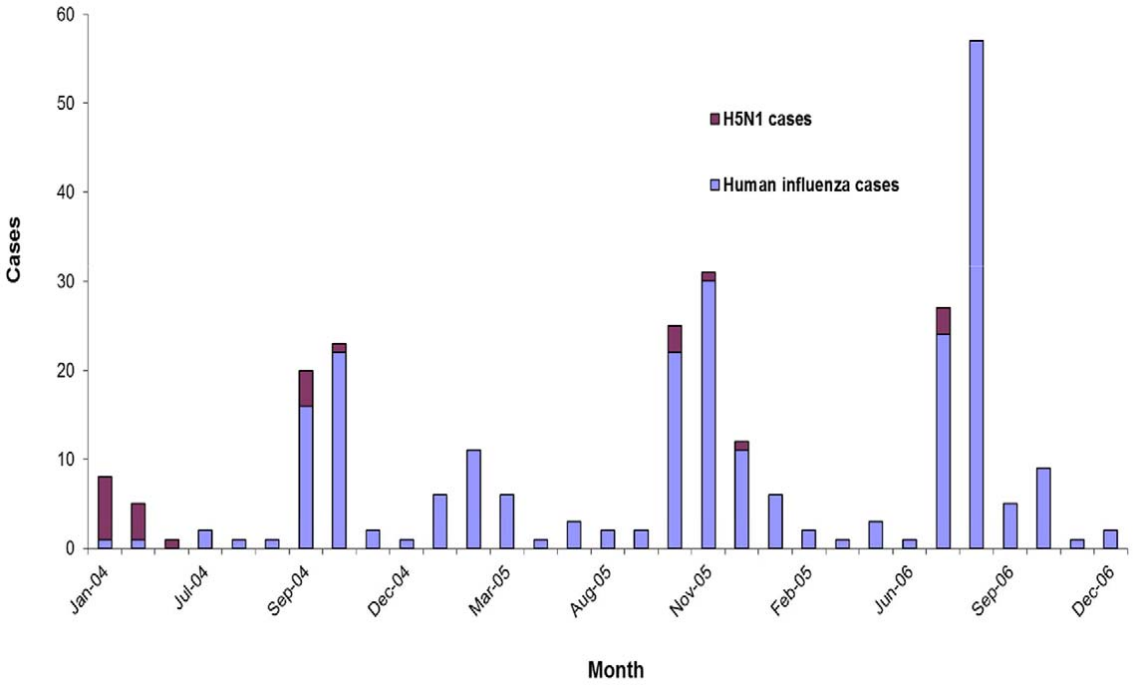
Seasonality of 25 patients with influenza A(H5N1) virus infection and a subset of 252 hospitalized patients with human influenza virus infection, Thailand, 2004–2006.

Although human influenza cases were detected in virtually every Thai province, H5N1 cases were geographically localized to the central and northern regions where most commercial poultry production was located. H5N1 cases were detected without a consistent seasonal pattern; whereas human influenza cases were most frequently detected during one or two annual periods of peak influenza activity occurring between July and November or between January and March (Figure 2) [5], [8].

Several differences related to H5N1 virus exposure were observed between H5N1 and human influenza cases (Table 1). H5N1 cases were significantly more likely to have one or more direct, potential high risk exposures to H5N1 virus, for example consuming sick or dead birds; none had as the only exposure the presence of sick or dead birds in the neighborhood (Table 1). Age, gender, and occupation were similar between the two groups.

**Table 1:** Demographic and epidemiologic characteristics of patients with influenza A(H5N1) infection and seasonal human influenza virus infection, National Avian Influenza Surveillance (NAIS), Thailand, 2004–2006.

		H5N1 Influenza	Human Influenza	
		(N = 25)	(N = 252)	
**Sex**	Male	16 (64)	147 (59)	
**Age**	Median, years (range)	18 (1.6–68)	14 (.08–102)	
	0–9 yrs	10 (40)	89 (25)	
	10–19 yrs	4 (14)	64 (25)	
	20–29 yrs	4 (16)	19 (8)	
	30–39 yrs	1 (4)	18 (7)	
	40–49 yrs	3 (12)	20 (8)	
	> = 50 yrs	3 (12)	42 (17)	
**Occupation**	Poultry worker	2 (8)	8 (3)	
	Healthcare worker	0 (0)	1 (<1)	
	Other	22 (92)	224 (96)	
**Potential H5N1 virus exposures**				
**Direct exposures**	Consumed sick or dead birds	**7 (39)**	**8 (5)**	*
	Touched sick or dead birds	**16 (70)**	**69 (32)**	*
	Cared for sick or dead birds	**9 (45)**	**44 (23)**	*
	Butchered sick or dead birds	**3 (17)**	**7 (4)**	*
	Sick or dead birds in household	**18 (85)**	**97 (50)**	*
	Contacted another human H5N1 case	3 (16)	7 (4)	
	≥1 direct exposures	**25 (100)**	**151 (60)**	*
	No direct exposure to H5N1	0 (0)	101 (40)	
**Indirect** ** **exposures**	Indirect exposure only	**0 (0)**	**38 (15)**	*
	Indirect plus ≥1 direct exposures	12 (48)	72 (29)	
**No known direct or indirect exposures**		**0 (0)**	**63 (25)**	*

*P<0.05.

**Indirect exposures: the presence of sick or dead birds in the patient's neighborhood.

During 2004, the first year of the surveillance, H5N1 cases presented to hospital later than did human influenza cases (median: 5 vs. 2 days, p<.01); this difference persisted during the subsequent two years of surveillance (2005–2006) (data not shown).

### Epidemiological and clinical characteristics of fatal and non-fatal H5N1 cases

Among H5N1 cases, those with fatal and non-fatal illness were similar with regard to age (median: 14 vs. 25 years old, p = .86), presence of an underlying medical condition, presenting symptoms, and radiographic evidence of pneumonia or ARDS on an admission chest X-ray (Table 2). Vomiting was more frequently observed among those with fatal illness, but was not statistically significant. However, several notable differences in admission laboratory testing values were observed between the two groups; fatal compared to non-fatal H5N1 cases had lower median white blood cell (WBC) counts, lower median lymphocyte and platelet counts, and higher mean serum AST and ALT levels.

**Table 2:** Chronic medical conditions, presenting signs and symptoms, laboratory testing, and chest X-ray results among persons with fatal and non-fatal influenza A(H5N1) virus infections.

	All H5N1 (N = 25)	Non-fatal H5N1 (N = 8)	Fatal H5N1 (N = 17)	p-value	UnadjustedOR [95% CI]*
**Chronic medical conditions**					
≥1 condition	4 (16)	2 (25)	2 (12)	0.57	0.4 [0.04–3.52]
**Signs and symptoms at presentation**					
Fever>38.2°C	23 (96)	6 (86)	17 (100)	0.29	**
Cough	20 (83)	6 (86)	14 (82)	1	1.29 [0.11–15.0]
Sore throat	9 (38)	2 (29)	7 (41)	0.67	0.57 [0.01–3.83]
Dsypnia†	17 (81)	4 (57)	13 (76)	0.37	0.41 [0.06–2.66]
Headache	2 (8)	0 (0)	2 (12)	1	**
Myalgia	6 (25)	1 (14)	5 (29)	0.63	0.4 [0.4–4.23]
Altered mental status§	2 (8)	0 (0)	2 (12)	1	**
Abdominal pain	4 (17)	1 (14)	3 (18)	1	**
Vomiting	12 (50)	1 (14)	11 (65)	0.07	0.09 [0.01–0.94]
Diarrhea	5 (21)	1 (14)	4 (23)	1	0.45 [0.05–5.94]
**Laboratory values**					
White blood cell (WBC) count	5.1 (1.1–18.3)	7.6 (3.1–13.6)	4.1 (1.1–18.3)	**0.05**	n/a
Neutrophil count	3.9 (1.2–15.6)	4.5 (1.6–8.9)	3.4 (1.2–15.6)	0.11	n/a
Lymphocyte count	1.2 (.1–4.3)	1.8 (.8–4.3)	.9 (.1–2.7)	0.**02**	n/a
Platelet count	172 (77–528)	254 (178–528)	152 (77–304)	**<.01**	n/a
Hemoglobin	12.3 (9–16.7)	10.4 (9–14)	12.7 (11–16.7)	**0.02**	n/a
AST	148 (17–1032)	27 (17–306)	181 (34–1032)	**0.05**	n/a
ALT	50 (7–1048)	20 (7–82)	55 (26–1048)	**0.04**	n/a
BUN	12 (4–61)	9 (4–9)	22 (7–61)	**0.05**	n/a
Creatinine	1 (.2–2.8)	0.7 (.2–.8)	1.4 (.5–2.8)	**0.03**	n/a
**Admission chest X-ray**					
Pneumonia¶	4 (50)	7 (41)	11 (44)	1	0.7[0.13–3.79]
Pneumonia¶ or ARDS	4 (50)	7 (41)	11 (44)	1	0.7 [0.13–3.79]
**Result of any chest X-ray**					
Pneumonia¶	15 (60)	4 (50)	11 (65)	0.67	1.83 [0.33–10.09]
Pneumonia¶ or ARDS	20 (80)	4 (50)	16 (94)	**0.02**	**16.0 [1.38–185.4]**
**Complications**					
Hypotension	13 (57)	0 (0)	13 (81)	**<.001**	**
Respiratory failure	17 (71)	0 (0)	17 (100)	**<.001**	**
ARDS	14 (56)	0 (0)	14 (82)	**<.001**	**
Need for ICU	17 (68)	0 (0)	17 (100)	**<.001**	**
**Key Intervals**					
Symptom onset to first medical care	4 (1–14)	4 (3–14)	3 (1–10)	0.2	n/a
Symptom onset to hospital admission	4 (2–18)	4.5 (3–18)	4 (2–10)	0.34	n/a
Symptom onset to oseltamivir treatment	7 (4–21)	7 (5–7)	8 (4–21)	0.39	n/a
Hospital admission to oseltamivir treatment	2.5 (0–14)	2 (0–3)	3 (0–14)	0.28	n/a
Hospital admission to death or discharge	n/a	13 (1–21)	6 (2–23)	n/a	n/a

N/a: not applicable;

*unadjusted OR and 95% CI of a fatal H5N1 outcome among H5N1 cases;

† dyspnea: defined as any recorded difficulty breathing or shortness of breath in any age group, or in a child less than age 5, the presence of stridor or chest in-drawing;

§ altered mental status: defined as any recorded presence of altered mental status, confusion, or unconsciousness;

¶ pneumonia: defined as the presence of alveolar infiltrates, interstitial infiltrates, or lobar consolidation on chest X-ray;

**not possible to calculate an unadjusted OR.

Similar proportions of fatal and non-fatal H5N1 cases received treatment with the antiviral agent oseltamivir (71% vs. 57%, p = .65), an antibiotic agent (100% vs. 100%, p = 1), and or a corticosteroid (29% vs. 43%, p = .65). Clinical complications noted in H5N1 cases were more frequently present among fatal compared to non-fatal cases, including respiratory failure requiring intubation, hypotension requiring inotropic agents, development of acute respiratory distress syndrome (ARDS), and need for intensive care unit (ICU) management (Table 2). Similar clinical intervals between symptom onset, hospitalization, and oseltamivir treatment were observed between fatal and non-fatal H5N1 cases. Non-fatal H5N1 cases were hospitalized for a median of 13 days (range, 1–21), while fatal H5N1 cases died of their illness within a median of 6 days after hospitalization (range, 2–23).

### Epidemiological and clinical characteristics of fatal H5N1 and fatal human influenza cases

Fatal human influenza cases compared to fatal H5N1 cases tended to be older (median age: 39 vs. 14 years, p = .14), although not statistically significant, and were significantly more likely to have a chronic medical condition (Table 3). Clinical symptoms at admission were similar, except for fever, sore throat, and vomiting which was significantly more common in fatal H5N1 cases. Similar proportions of cases in both groups experienced shortness of breath. A few admission laboratory testing values differed between the groups; fatal H5N1 cases compared to fatal human influenza cases had lower median white blood cell (WBC) counts, lower median neutrophil counts, and a lower median lymphocyte count (although the latter did not achieve statistical significance). Fatal cases in both groups had similar (low normal) median values for platelet counts and mild to moderate elevations in serum liver enzymes levels (AST and ALT). The initial chest X-ray at admission was more likely to show radiographic evidence of pneumonia or ARDS among fatal human influenza cases compared to fatal H5N1 cases; however, these differences did not persist if radiographs from the entire hospital course were considered. Overall, fatal H5N1 cases were significantly more likely to develop ARDS during the course of illness compared to fatal human influenza cases.

**Table 3:** Comparison of clinical characteristics between persons with fatal influenza A(H5N1) virus infection and fatal human influenza virus infection.

	Fatal H5N1	Fatal Human Influenza	p-value	UnadjustedOR [95% CI]*
	(N = 17)	(N = 22)		
**Chronic medical conditions**				
≥1 condition	2 (12)	15 (68)	**0.001**	**0.06 [0.01–0.35]**
**Sign and Symptoms Presentation**				
Fever>38.2°C	17 (100)	9 (41)	**<.001**	**
Cough	14 (82)	15 (68)	0.46	0.46 [0.1–2.13]
Sore throat	7 (41)	1 (5)	**0.01**	**0.07 [0.01–0.63]**
Dsypnia†	13 (76)	16 (73)	1	0.82 [0.19–3.54]
Headache	2 (12)	0 (0)	0.18	**
Myalgia	5 (29)	1 (5)	**0.07**	**0.11 [0.11–1.01]**
Altered mental status§	2 (12)	6 (27)	0.37	0.36 [0.06–2.04]
Abdominal pain	3 (18)	0 (0)	0.07	**
Vomiting	11 (65)	4 (18)	**<.01**	**0.12 [0.03–0.53]**
Diarrhea	4 (23)	2 (9)	0.37	0.33 [0.05–2.04]
**Laboratory values**				
White blood cell (WBC) count	4.1 (1.1–18.3)	8.7 (0.9–34.7)	**0.01**	n/a
Neutrophil count	3.4 (1.2–15.6)	6.0 (.05–29.5)	**0.02**	n/a
Lymphocyte count	.9 (.1–2.7)	1.5 (.4–5.0)	0.09	n/a
Platelet count	152 (77–304)	156 (90–392)	0.61	n/a
Hemoglobin	12.7 (11–16.7)	12.2 (8.5–17.7)	0.41	n/a
AST	181 (34–1032)	161 (48–2289)	0.88	n/a
ALT	55 (26–1048)	70 (18–444)	0.47	n/a
BUN	22 (7–61)	25 (7–92)	0.46	n/a
Creatinine	1.4 (.5–2.8)	1.3 (.2–10.4)	0.81	n/a
**Admission Chest X-ray**				
Pneumonia¶	7 (41)	18 (82)	**0.02**	**0.15 [0.04–0.66]**
Pneumonia¶ or ARDS	7 (41)	19 (86)	**<.01**	**0.11 [0.02–0.52]**
**Any Chest X-ray**				
Pneumonia¶	11 (65)	18 (82)	0.28	0.4 [0.09–1.77]
Pneumonia¶ or ARDS	16 (94)	19 (86)	0.62	2.53 [0.24–26.72]
**Complications**				
Hypotension	13 (81)	20 (91)	0.63	2.3 [0.34–15.75]
Respiratory failure	17 (100)	22 (100)	n/a	n/a
ARDS	14 (82)	7 (32)	**<.001**	**0.1 [0.02–0.46]**
Need for ICU	17 (100)	15 (68)	**0.01**	n/a
**Key intervals**				
Symptom onset to first medical care	3 (1–10)	2 (0–7)	0.22	n/a
Symptom onset to hospital admission	4 (2–10)	2 (0–7)	**0.02**	n/a
Symptom onset to oseltamivir treatment	8 (4–21)	4 (4–7)	**0.02**	n/a
Hospital admission to oseltamivir treatment	3 (0–14)	1 (0–3)	0.1	n/a
Hospital admission to death or discharge	6 (2–23)	3.5 (0–68)	0.09	n/a

N/a: not applicable;

*unadjusted OR and 95% CI of a fatal H5N1 outcome versus a fatal human influenza outcome;

† dyspnea: defined as any recorded difficulty breathing or shortness of breath in any age group, or in a child less than age 5, the presence of stridor or chest in-drawing;

§ altered mental status: defined as any recorded presence of altered mental status, confusion, or unconsciousness;

¶ pneumonia: defined as the presence of alveolar infiltrates, interstitial infiltrates, or lobar consolidation on chest X-ray;

**not possible to calculate an unadjusted OR.

Among the two groups, fatal H5N1 cases were more likely to receive oseltamivir that fatal human influenza cases (71% vs. 23%, p<0.01). However, fatal H5N1 cases experienced greater delays in the number of median days between symptom onset and hospital admission, and even more significant delays between symptom onset and oseltamivir treatment (median: 8 vs. 4 days, p<.02)(Table 3).

## Discussion

During the 2004–2006 influenza A(H5N1) epizootic in Thailand, we identified few differences in admission clinical or epidemiologic characteristics between fatal and non-fatal H5N1 cases; however, the presence of laboratory abnormalities in white blood cell counts and liver enzymes, hypotension, or ARDS may suggest a poor prognosis. Compared to patients hospitalized for human influenza infections, all H5N1 cases had a history of direct exposure to sick or dead birds. H5N1 cases had a high case fatality ratio in Thailand, unlike patients with human influenza infection identified from NAIS. However, severely ill patients with human influenza infection who subsequently died looked clinically similar to patients with H5N1 infection. The presence of underlying medical conditions, lack of measured fever, normal WBC counts, and the absence of ARDS were the best predictors in severely ill patients with suspect H5N1 that the infection was due to human influenza viruses versus H5N1 virus.

Among H5N1 cases, previous reports have described several admission findings associated with fatal outcome that might help clinicians risk stratify patients. Among H5N1 cases in Thailand during 2004–2006, we found that most admission signs and symptoms, chest radiograph findings, and epidemiologic factors were not useful in estimating risk for fatal disease. However, multiple laboratory abnormalities on admission were associated with a fatal outcome among H5N1 cases. Similar to other reports, we found that fatal H5N1 cases compared to those who survived, were more likely to have a lower median white blood cell count (WBC) or leukopenia [9], [10], lower median lymphocyte count or lymphopenia [10], lower median platelet count or thrombocytopenia [9], [10], [11], elevated serum liver enzymes (AST and ALT) [9], [10], [11], and elevated serum BUN and creatinine levels [9]. We do not have the power to determine specific cut-off values for these laboratory tests that might predict a poor prognosis. Also, fatal compared to non-fatal H5N1 cases were more likely to have circulatory and respiratory failure [10], [11], need for ICU management, and progression to ARDS [11]. There were no significant differential delays between symptom onset and clinical care or treatment among the two groups in our study that might influence our results. Although vomiting was more common among fatal H5N1 cases in our series, a study by Liem et al reported diarrhea, not vomiting, to be associated with fatal H5N1 outcome. [10]. We observed no significant differences in the proportions of cases presenting with diarrhea. The presence of laboratory abnormalities in WBC counts and liver enzymes, hypotension, and progression to ARDS likely suggest a poor prognosis for patients with influenza A (H5N1) virus infection.

We were able to directly compare patients infected with influenza A (H5N1) virus or human influenza viruses identified from the same surveillance system. Several epidemiologic factors may offer clues that the infection is a human influenza infection versus H5N1 virus infection. Human influenza viruses were most common during annual peaks in human influenza circulation, while H5N1 cases had no seasonality. Also, H5N1 cases had a history of direct exposure from dead or sick birds and were from provinces with commercial poultry operations; many patients with human influenza infection had only indirect exposure to dead or sick birds. Finally, underlying medical conditions that are associated with complications (including death) from human influenza infection were present in less than 15% of fatal H5N1 cases. In contrast, almost 60% of fatal human influenza infections had chronic medical conditions. These differences may offer some clues when laboratory testing is delayed or lacking to target control efforts to identify and prevent secondary human transmission H5N1 cases during a large epizootic outbreak.

Clinically, patients with severe human influenza infections that resulted in death were generally similar to patients with influenza A (H5N1) infection; shortness of breath was commonly observed on presentation and platelet counts and liver enzymes levels on initial laboratory assessment were similar. Also, similar proportions of cases in both groups had disease progression complicated by circulatory and respiratory failure. Although these clinical characteristics have been previously described in large series of severe and fatal H5N1 cases [7], [9], [10], [11], [12], [13], we found that they were not unique to H5N1 cases. The lack of underlying medical conditions, presence of a measured fever and low WBC counts, and the presence of ARDS were the best predictors that the infection was H5N1 virus versus human influenza virus among severely ill patients identified by NAIS.

Although the NAIS system was developed to find H5N1 cases, it eventually detected 80 times more human influenza cases [3], primarily as a consequence of three factors: 1) the ubiquitous nature of poultry exposures amongst the surveillance population, 2) overlapping clinical respiratory syndromes, and consequently non-specific case definitions, and 3) a comprehensive and specific laboratory-based molecular diagnostic testing of all suspect H5N1 cases. In our study cohort, all confirmed H5N1 cases had at least one documented direct H5N1 exposure; in contrast, among those testing positive for human influenza, 40% had no documented direct H5N1 exposure (overall, 15% had only indirect exposures, and 25% had no direct or indirect exposure). All laboratory confirmed H5N1 cases met the broad clinical case definition of a suspected H5N1 case disseminated by the MOPH. However, had a more narrow WHO suspect H5N1 case definition been adopted, four fatal and four non-fatal H5N1 cases might have been missed due to the initial absence of documented shortness of breath as required under the WHO suspect H5N1 case definition. Although using a broader surveillance case definition was resource intensive, the MOPH case definition was nonetheless a reasonable approach for case ascertainment given the risks of misdiagnosing and delaying treatment for a highly fatal H5N1 infection. Lastly, as noted in previous studies, we observed delays in care between onset of H5N1 symptoms and medical attention or initiation of antiviral therapy, especially among H5N1 cases and fatal human influenza cases [5], [7], [9], [11], [12], [13], [14], [15], [16], [17], [18], [19]. The benefit of oseltamivir is greatest early in the disease course for both severe infections due to either H5N1 or human influenza [7]. Decentralization (to the extent possible) of access to oseltamivir and the promotion of clinical awareness of severe influenza virus infections and the benefits of early antiviral treatment among frontline clinicians might improve clinical outcomes for many patients with suspect H5N1 infection during an influenza A (H5N1) epizootic.

Our study was subject to several limitations. NAIS was a passive surveillance system, and H5N1 cases may have been missed as a consequence of under-reporting and or under-detection. In addition, the characteristics of the patients with human influenza infection from NAIS are different from those identified from surveillance for human influenza infections. Consequently, adults ≥65 years of age and very young children with human influenza, with no poultry exposure, were not represented. Clinical and epidemiologic data on individual cases varied according to the completeness of medical records. Also, information on potential H5N1 exposures were extracted retrospectively and may have been more complete for confirmed H5N1 cases. Due to our small sample size, we may not be able to detect some differences in clinical characteristics between fatal and non-fatal H5N1 cases and human influenza cases. Finally, inferences regarding the clinical presentation of H5N1 cases are limited to the clade one H5N1 viruses circulating in Thailand and the surrounding region during the study period and may not be representative of other clades of H5N1 virus known to cause human disease in other parts of the world.

As a result of Thailand's strong public health response to the emergence of influenza A(H5N1) virus infection in humans, we were provided a rare opportunity to compare H5N1 cases and human influenza cases identified by the same surveillance system. In lieu of laboratory diagnostics, history of direct exposure to dead or sick birds and lack of underlying medical conditions that are associated with complications due to human influenza may provide clues for targeting H5N1 control efforts. Fatalities from human influenza infection were uncommon. However, patients with severe human influenza infection had a clinical presentation that overlapped considerably with H5N1 cases. All of these patients, those with H5N1 infection and those with severe human influenza infection, would benefit from early empiric antiviral treatment.
